# Predictors of Root Resorption in Lateral Incisors Adjacent to Maxillary Impacted Canines in CBCT Images: A Retrospective Study

**DOI:** 10.1002/cre2.70381

**Published:** 2026-06-07

**Authors:** Mahkameh Moshfeghi, Maryam Hosseini, Parastoo Iranparvar, Amirali Momeni, Negin Eslamiamirabadi, Mina Biria

**Affiliations:** ^1^ Department of Oral and Maxillofacial Radiology, School of Dentistry Shahid Beheshti University of Medical Sciences Tehran Iran; ^2^ School of Dentistry Shahid Beheshti University of Medical Sciences Tehran Iran; ^3^ Department of Pediatric Dentistry, School of Dentistry Shahid Beheshti University of Medical Sciences Tehran Iran; ^4^ Faculty of Dentistry Universite de Montreal Montreal Canada

**Keywords:** cone‐beam computed tomography, cuspid, impacted tooth, root resorption

## Abstract

**Objectives:**

This study aimed to assess the predictive factors of root resorption in lateral incisors adjacent to impacted maxillary canines using cone‐beam computed tomography.

**Material and Methods:**

In this retrospective descriptive‐analytic study, 150 samples of impacted canines from 138 CBCT images were collected from the files of 12–35‐year‐old patients. The association between the severity and location of root resorption in lateral incisors adjacent to impacted maxillary canines and the patients’ age, sex, impacted canine's angulation, position, and follicle size was evaluated. Position and angulation of the impacted teeth were measured using the OnDemand3D software. Kruskal–Wallis test, Fisher's exact test, Mann–Whitney test, Kolmogorov–Smirnov test, Spearman's rank correlation coefficient, and multinomial logistic regression were used for data analysis.

**Results:**

The probability of resorption in the middle‐third of the roots of lateral incisors decreased by 20% with each millimeter of increase in the distance between the impacted canines’ cusp tip from the occlusal plane (*p* = 0.009). There was a significant association between the severity of lateral incisors’ root resorption and sex; root resorption was significantly more severe in females (*p* = 0.029).

**Conclusions:**

Among the evaluated variables, the vertical position of the impacted canine influenced the location of the lateral incisors’ root resorption. The severity of root resorption was higher in females.

## 1 Introduction

Impaction of a permanent tooth usually occurs due to the tooth's failure to move and reach its correct position in the dental arch during the eruption period (Ali et al. 2021; Dağsuyu et al. 2017). Maxillary canine impaction is the second most common eruption failure, affecting 1%–3% of the population (Alqerban et al. 2016), and can lead to pathological problems (Ali et al. 2021; Rafflenbeul et al. 2019). The most important irreversible adverse effect of maxillary canine impaction is the root resorption of its adjacent teeth, especially the lateral incisor, which can lead to lateral incisor tooth loss (Kalavritinos et al. 2020). To date, several risk factors have been identified with regard to the root resorption of lateral incisors, while the causal factor remains unknown (Cuminetti et al. 2017).

Early diagnosis of root resorption may not be possible, as it is often asymptomatic; even though resorption can reach the dental pulp, no clinical symptoms may be evident. Consequently, the lesion is usually diagnosed when the tooth needs to be extracted (Ali et al. 2021; Alqerban et al. 2016). Due to the rapid progress of root resorption of teeth adjacent to impaction, identifying teeth with a high risk of root resorption is essential for early intervention and treatment (Rafflenbeul et al. 2019). Knowledge regarding the effects of maxillary canine impaction on the adjacent lateral root resorption (ALRR) and awareness of root resorption risk factors can provide efficient clinical guidance for clinicians to determine the prognosis, treatment planning, and the appropriate timing for intervention (Patel and Saberi 2018).

Two‐dimensional imaging approaches such as periapical, panoramic, and occlusal radiographs can be helpful with the diagnosis and follow‐up of tooth eruption conditions and evaluating treatment results. However, they have limitations such as dimensional distortions and superimpositions, which may render them inadequate for diagnosis and treatment in most cases (Sarica et al. 2019). Root resorption can be immediately identified using three‐dimensional imaging techniques such as cone beam computed tomography (CBCT) (Cuminetti et al. 2017). CBCT radiographs have higher sensitivity and accuracy in the diagnosis of root resorption compared to two‐dimensional radiographic techniques (Kalavritinos et al. 2020). Studies have indicated that resorption of roots with a diameter of less than 0.6 mm and a depth of less than 0.3 mm cannot be easily identified in two‐dimensional radiographic images, especially if the impacted tooth is buccally positioned (Cuminetti et al. 2017). Moreover, CBCTs can provide three‐dimensional details regarding the position of the impacted maxillary canine (IMC) and the adjacent lateral incisor, their position towards the floor of the nasal foramina, maxillary sinus, and mandibular canal, and these details can be observed from different angles (Sarica et al. 2019; Schroder et al. 2018; Ucar et al. 2017).

The association between ALRR and some variables, including the position and angle of the impacted tooth, patient's sex and age, the distance between IMC and the midline, and the occlusal plane, as well as the IMC's follicle size, has been evaluated in previous studies (Ali et al. 2021; Dağsuyu et al. 2017; Ardakani et al. 2021). However, none of the previously conducted studies have evaluated all these variables in a single population. Moreover, no study has yet been conducted that would evaluate all these variables in a single Iranian population with an adequate sample size.

This study aimed to evaluate the association between the abovementioned factors and ALRR in a sample of the Iranian population. The objectives included observing any association of IMC position, follicle size, and patients’ age and sex, with ALRR. The null hypothesis was that there would be no significant association between any of the studied factors and ALRR. The results of this study can help clinicians to make early interventions and appropriate treatment plans to prevent ALRR in patients with IMC.

## 2 Methods and Materials

The study was conducted after its experimental protocol was approved by the Committee for Ethics in Research, School of Dentistry, Shahid Beheshti University of Medical Sciences (IR.SBMU.DRC.REC.1398.198). The sample size of this retrospective descriptive‐analytic study was calculated as 150 according to the formula (
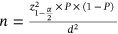
) with a confidence interval of 95% ($z_{1-\frac{\alpha }{2}}$) = 1.96 and a *P* (impaction prevalence) = 0.4 (Dağsuyu et al. 2018) and an estimation error of *d* = 0.08.

Data were collected using the documents in the CBCT archive of the department of oral and maxillofacial radiology, Faculty of Dentistry, Shahid Beheshti Medical University, Tehran, Iran, from September 2018 to May 2020. Patients between 12 and 35 years of age who had taken CBCT images due to different diagnostic reasons and had at least one IMC adjacent to a lateral incisor were included in this study. The presence of a maxillary first molar to determine the occlusal plane was among the inclusion criteria. The exclusion criteria included patients under orthodontic treatment, having a pulp‐treated lateral incisor adjacent to IMC, having a craniofacial anomaly, hereditary syndromes, systemic diseases that might affect tooth and bone growth and formation, and low‐quality radiographic images (Hadler‐Olsen et al. 2015).

CBCT images of 138 patients (including 150 MICs), taken with standard resolution and field of view (FOV) of 12 × 8 mm with a voltage of 110 kVp obtained through the NewTom VGI device (QR, Verona, Italy), were evaluated in this study. Data were collected through patient records and observation and measurement of their CBCT images using the OnDemand3D software. Observation and measurement of CBCT images were conducted by a senior dental student at Shahid Beheshti Dental School, under the supervision of an experienced associate professor of the oral and maxillofacial radiology department, by whom she had received specific training for observing, diagnosing, and analyzing CBCT images. To ensure the reliability of data, the intra‐observer agreement was first tested on 30 samples with an interval of 21 days.

**Figure 1:**
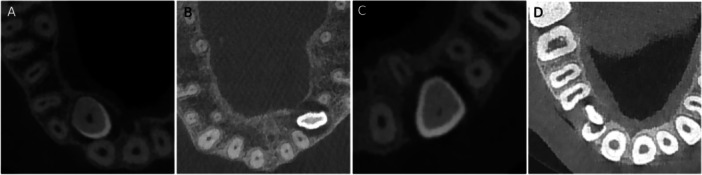
Axial view of root resorption.

In this research, age and gender were recorded from patients’ documents. The Kurol–Ericson scale was used for measuring the severity of ALRR. This scale has 4 categories, from “no resorption” to “severe resorption.” (A) Grade 1 = no root resorption; (B) Grade 2 = slight root resorption (up to half of the dentinal width); (C) Grade 3 = moderate root resorption (half or more of the dentinal width, not involving the dental pulp); and (D) Grade 4 = severe root resorption (with dental pulp involvement) (Figure 1) (Dağsuyu et al. 2018).

**Figure 2:**
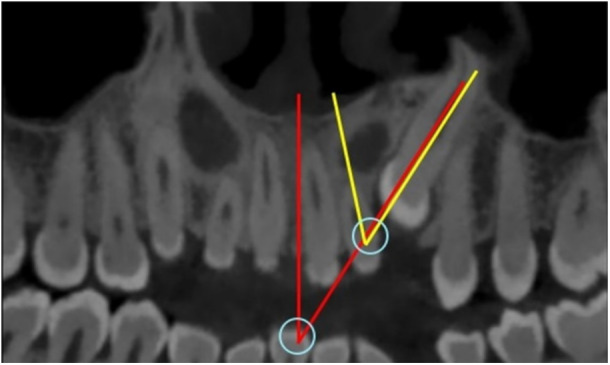
IMC position in coronal view.

The angle between the long axis of IMC and the adjacent lateral tooth (CAL) (yellow) and the midline (CAM) (red) in the coronal views were obtained from CBCTs (Figure 2).

**Figure 3:**
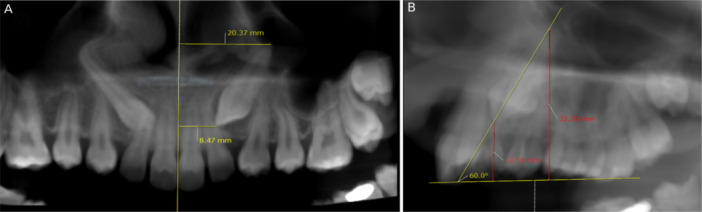
IMC location in coronal and sagittal views. (A) The distance between the cusp tip and the root apex from the midline in the coronal view, and (B) the distance of the same points from the occlusal plane and the angle between the long axis of IMC and the occlusal plane (CAO) in the sagittal view.

The software enabled the evaluation of 3D images in coronal and sagittal planes. The transverse and vertical position and the distance between the cusp tip and apex of IMC and the midline were recorded in the coronal plane. The angle between the long axis of IMC and the occlusal plane (CAO) was measured in the sagittal plane using the software (Figure 3).

**Figure 4:**
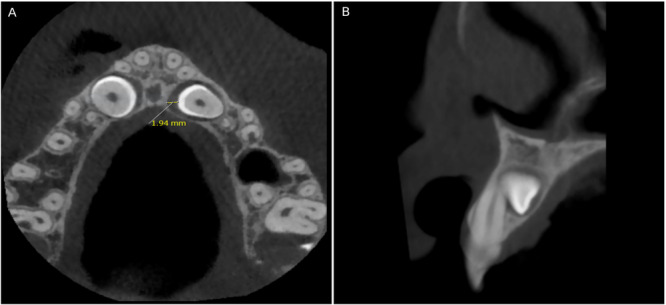
Follicle size of IMC and it's relation with ALRR. (A) Evaluating IMC's follicle size and the severity of ALRR in the axial view; (B) evaluating the severity of ALRR and the sagittal position of the IMC in relation to the adjacent lateral incisor in the cross‐sectional view.

Using the images in the axial view, the size of the canine dental follicle and the severity of ALRR were evaluated. The severity of lateral incisor root resorption and buccal/palatal position of IMC were also evident in sagittal images, which helped to ensure the accuracy of the diagnosis (Figure 4). The position of IMC in these sections was either buccal, palatal, or bicortical (also described as intermediate, central, or arch‐aligned) (Mejía‐Milian et al. 2022).

Kolmogorov–Smirnov test was used to evaluate the normal distribution of continuous data, and the intraclass correlation coefficient (ICC) was used to evaluate the reliability of continuous variables. Reliability scores higher than 0.75 were considered excellent agreement according to Rosner's division (Rosner 1982). Kappa coefficient was used to evaluate the reliability of categorical data, and a kappa ≥ 0.6 was considered acceptable (Seigel et al. 1992). To evaluate the effect of the independent variables on the location of ALRR, Fisher's exact test and multinomial logistic regression were used. Kruskal–Wallis test, Mann–Whitney test, and Spearman's rank correlation coefficient were used to evaluate the association of continuous and categorical variables with the severity of root resorption. Type 1 error was set at *α* = 0.05, so *p*‐ value < 0.05 was considered significant. The SPSS software version 20 was used for statistical analysis.

## 3 Results

In this retrospective study, 150 CBCT images of 112 female (74.7%) and 38 male (25.3%) patients with an average age of 24.16 ± 8.00 years were evaluated; 24 IMCs (16%) were buccally, and 126 (84%) were palatally positioned. In 23 patients (15.3%), the vertical position of the IMC towards the adjacent lateral incisor roots was in the apical third, in 83 samples (55.3%) in the middle third, and in 44 patients (29.3%) in the incisal third. Degrees of 1, 2, 3, and 4 of ALRR had an incidence of 109 (72.9%), 20 (13.1%), 10 (6.7%), and 11 (7.3%), respectively. With regards to the resorption location, in 18 patients (12%), resorption was located in the apical third, in 22 patients (14.7%) in the middle third, and only in one patient (0.7%) in the incisal third of the root.

### 3.1 Intra‐Observer Agreement

Kolmogorov–Smirnov test revealed the normal distribution of continuous variables (*p* > 0.05). Therefore, the ICC reliability coefficient was used to evaluate the reproducibility of the data. All ICC reliability coefficients were higher than 0.75 (range: 0.87–0.99), which was reported as excellent agreement according to Rosner's division (Rosner 1982). For all five variables of sagittal, vertical, and transverse canine position, severity, and location of resorption, the kappa coefficient was > 0.8.

### 3.2 Sex, Age, and Follicular Size

The results indicated a higher severity of ALRR in females (Table 1). There was no significant association between the location of ALRR and the patients’ sex (Table 2).

**Table 1:** Distribution of degrees of root resorption severity according to impacted canine position and sex (Kruskal–Wallis and Mann–Whitney *U* test).

		Resorption degree				Total	*p*‐value
		1	2	3	4		
Vertical position	Apical	17 (73.9%)	3 (13.0%)	1 (4.3%)	2 (8.7%)	23	0.29
	Middle	56 (67.5%)	14 (16.9%)	7 (8.4%)	6 (7.2%)	83	
	Occlusal	36 (81.8%)	3 (6.8%)	2 (4.5%)	3 (6.8%)	44	
Sagittal position	Labial	16 (66.7%)	3 (12.5%)	3 (12.5%)	2 (8.3%)	24	0.43
	Palatal	93 (73.8%)	17 (13.5%)	7 (5.6%)	9 (7.1%)	126	
Transverse position	Mesial	84 (71.2%)	16 (13.6%)	9 (7.6%)	9 (7.6%)	118	0.42
	Distal	25 (78.1%)	4 (12.5%)	1 (3.1%)	2 (6.3%)	32	
Sex	Female	76 (67.8%)	18 (16.2%)	9 (8.1%)	9 (8.1%)	112	0.03
	Male	33 (86.8%)	2 (5.3%)	1 (2.6%)	2 (5.3%)	38	

**Table 2:** Distribution of location of root resorption according to the impacted canine position and sex (Fisher's exact test).

		Resorption location			Total	*p*‐value
		No resorption	Apical	Middle		
Vertical position	Apical	17 (77.3%)	0 (0%)	5 (22.7%)	22	0.09
	Middle	56 (67.5%)	13 (15.7%)	14 (16.9%)	83	
	Incisal	36 (81.8%)	5 (11.4%)	3(6.8%)	44	
Sagittal position	Labial	16 (66.7%)	5 (20.8%)	3 (12.5%)	24	0.36
	Palatal	93 (74.4%)	13 (10.4%)	19 (15.2%)	125	
Transverse position	Mesial	84 (71.8%)	16 (13.7%)	17 (14.5%)	117	0.58
	Distal	25 (78.1%)	2 (6.3%)	5 (15.2%)	32	
Sex	Female	76 (68.4%)	16 (14.5%)	19 (17.3%)	111	0.08
	Male	33 (86.8%)	2 (5.3%)	3 (7.9%)	38	

*Note:* Since root resorption in the incisal one‐third was observed in only one sample, this case was excluded, and the number of samples was considered 149 for this table.

In addition, no significant association was observed between the variables of age and canine follicle size with the severity and location of ALRR (*p* > 0.05), as shown by the results of the Spearman's rank correlation coefficient test.

### 3.3 Transverse, Sagittal, and Vertical Position

There was no significant association between the severity of ALRR and transverse, sagittal, and vertical position of IMC (Table 1). In addition, no significant relationship was found between the transverse, sagittal, and vertical position of IMC and the location of ALRR (Table 2).

### 3.4 Impacted Canine Distance to the Midline and Occlusal Plane

According to the results of the Spearman's rank correlation coefficient test, no significant relationship was observed between the severity of ALRR and the distance from the cusp tip and apex of IMC to the occlusal plane and to the midline (*p* > 0.05).

The distance between the canine cusp tip and the occlusal plane was significantly associated with the location of ALRR (*p* = 0.009). In other words, each millimeter of increase in the distance between the cusp tip of IMC and the occlusal plane decreased the probability of resorption in the middle third of the lateral incisor's root by 20% (OR = 0.777, 95% CI: [0.643–0.937]).

Evaluation of the association between the distance of the apex and the cusp tip of IMC from the midline and the location of ALRR indicated no significant statistical association (*p* > 0.05).

### 3.5 Impacted Canine Angulation to the Midline, Occlusal Plane, and Lateral Tooth Long Axis

No significant association was observed between the severity of ALRR and the angle between the long axis of IMC and the lateral incisor (CAL), the occlusal plane (CAO), and the midline (CAM) (*p* > 0.05), as shown by the results of the Spearman's rank correlation coefficient test. In addition, no relationship was found between the location of ALRR and the above‐mentioned angles.

## 4 Discussion

Early diagnosis of maxillary canine impaction and its associated factors is crucial for preventing its consequent adverse effects. The risk factors are either local or systemic. Local risk factors include lack of space in the dental arch, trauma, ankylosis of primary teeth, ectopic tooth bud, inflammation, pathologic lesions, and mesial movement of the adjacent teeth, all of which may lead to premature loss of these teeth. Systemic risk factors include malnutrition, anemia, rickets, vitamin D deficiency, endocrine diseases, syndromes such as Turner (Russell 2001), and specific infections such as syphilis and tuberculosis (Sarica et al. 2019). Genetic factors can also have a role in lateral incisor root resorption (Sarica et al. 2019). In our perspective, a comprehensive article about predictors of root resorption in lateral incisors adjacent to maxillary impacted canines on Iranian population has not yet been conducted.

In our study, CBCT images of 138 patients with 150 MICs were evaluated; 12 patients had bilateral maxillary canine impaction. The female‐to‐male ratio was 3:1. Studies of Kanavakis et al. (2015), Dagsuyu et al. (2017), Alqerban et al. (2016), and Andresen et al (2022) were in line with this study, indicating that the impaction of maxillary canine is more common in females. It is speculated that variations in genetics or craniofacial growth patterns between males and females may be the cause for the aforementioned difference (Dağsuyu et al. 2017; Russell 2001). However, female patients being more attentive to aesthetics and more willing to receive orthodontic treatment can also be the reason for this difference (Ardakani et al. 2021; Moshfeghi et al. 2013).

According to Alqerban et al. (2016), Guarnieri et al. (2016), and Ucar et al. (2017), no significant association was observed between sex and the location of root resorption, which is in accordance with our study. Moreover, these studies revealed that in cases with ALRR, the severity of resorption in females was significantly higher, which is in line with our results (Cuminetti et al. 2017). An explanation for the more severe ALRR in females could be the larger sample size of females in the study, resulting from the higher prevalence of maxillary canine impaction in females.

Since the delayed eruption of maxillary canines is usually noted after the age of 11 (Ericson and Kurol 1986) and the probability of root resorption in teeth adjacent to an impacted tooth increases with time (Cuminetti et al. 2017), the age range of our samples was limited to 12–35 years. In accordance with studies of Dogramaci et al. (2015), Rafflenbeul et al. (2019), and Kalavritinos et al. (2020), our study showed no association between the patients’ age and the severity and location of ALRR).

Hadler‐Olsen et al. (2015) reported that most of the ectopic canines were located palatally to the dental arch, whereas most of the normal canines were located in line with the dental arch. Brosson and Naoumova (2020) conducted a prospective longitudinal study on impacted canine and stated that in most cases, they were positioned palatally. The results of these studies are consistent with the results of the current study regarding the location of the IMC. Buccal impaction is usually related to insufficient space in the dental arch, while palatal impaction may occur even with adequate space (Ardakani et al. 2021).

Sun et al. (2024) evaluated the effect of bucco‐palatal incisor root inclination and its effect on ALRR and concluded that a close contact between the crown of palatal MIC and the root of the lateral incisor can result in a more buccally inclined lateral incisor root. Therefore, in such cases, the pressure from the MIC crown could result in a movement of the lateral incisor root. In cases of ALRR with palatal MIC, the lateral incisor had more lingual inclination than cases without ALRR, indicating that movement in lateral incisor roots could mitigate the risk of ALRR in some cases (Sun et al. 2024). A more general justification in this regard might be the fact that during the ectopic eruption process, palatal and buccal canines often continue their initial eruption path in the palatal or buccal direction, respectively. This movement, avoiding the lateral incisor, can help mitigate ALRR in some cases.

Rafflenbeul et al. (2019) evaluated root resorption of teeth adjacent to untreated IMCs. Results of that study showed that approximately half of the IMC (s) adjacent to the central and lateral incisors were located at the apical third, whereas the IMC close to the first and second premolars was located at the cervical third of their roots (Rafflenbeul et al. 2019). In this study, the highest prevalence of the vertical position of IMC towards the adjacent lateral incisor was in the middle third (55.3%), incisal third (29.3%), and apical third (15.3%), respectively. These results are in accordance with the study of Andresen et al. (2022).

Among the 150 samples evaluated in this study, 109 (72.9%) of adjacent lateral incisors had no root resorption, 20 (13.1%) showed slight, 10 (6.7%) moderate, and 11 (7.3%) severe root resorption. This is in accordance with Liu et al.'s (2008) study, in which 210 impacted canines were evaluated, and the prevalence of maxillary lateral root resorption was 27.2%. Strbac et al.'s (2013) study evaluated the prevalence of root resorption of maxillary incisors caused by IMCs and obtained similar results.

In accordance with the studies of Yan et al. (2012), Guarnieri et al. (2016), and Rafflenbeul et al. (2019), in our study, the rate of ALRR was higher in the middle (14.7%), apical (12%), and incisal third (0.7%), respectively. Yan et al. (2012) investigated potential risk factors for impacted canine‐associated root resorption in Chinese patients and concluded that the vertical position of IMC was not among the important variables for the degree of root resorption. Cuminetti et al.'s (2017) study led to the conclusion that the severity of resorption of the lateral incisor is not linked to the vertical position of IMC. These studies support the conclusion of the present study.

The results of our study showed that the IMC's follicle size was not significantly associated with the severity and location of ALRR. This finding is in accordance with the results of many previous studies, such as Cuminetti et al. (2017), Rafflenbeul et al. (2019), and Kalavritinos et al. (2020) studies. In fact, the follicular tissue can act as a barrier that prevents direct contact between the canine enamel and the adjacent incisor cementum. Histologic investigations indicate that, during normal eruption, the canine's dental follicle may uncover the root surface of the neighboring incisor by PDL resorption, without causing resorption of its hard tissue (Alqerban et al. 2009). Therefore, in a recent study by Alshawy and Kolarkodi (2023), even the highest canine dental follicle width was observed in cases of no or mild incisor root resorption. They concluded that other factors may be responsible for the root resorption more than the follicle size, such as the proximity, direction, and position of the MIC. Turker et al. (2025) most recently observed other responsible factors in adjacent root resorption due to canine impaction, such as dental follicle symmetry. In their research, follicle size or shape (symmetry) did not significantly affect resorption; however, the presence of a contact between the impacted canine and the adjacent root had the most significant effect (Türker et al. 2025). They mentioned the potential role of enzymes and cytokines inside the follicle in this process, while such molecular details need to be further evaluated in histologic and biologic studies, and radiographic studies do not reflect these mechanisms. They finally justified the conflicting results of different studies by differences in measurement criteria, voxel size, field of view, and demographic characteristics of samples, suggesting further standard investigations (Türker et al. 2025).

Ucar et al. (2017) reported that neither the transverse position of IMC nor the distance between the cusp tip and apex of IMC from the midline were associated with the severity and location of ALRR, which is in line with the findings of the present study.

Cuminetti et al. (2017) showed that the mean value of CAM was lower in patients with severe ALRR (1). However, similar to our study, Cuminetti et al. (2017) found no significant association between the transverse position of IMC and ALRR.

Also, our study showed no significant relationship between the sagittal position of IMC and the severity and location of ALRR. This finding is in line with the studies of Jawad Ucar et al. (2017), Hadler‐Olsen et al. (2015), and Jawad et al. (2016). The findings of Ardakani et al.'s (2021) study are, however, at odds with our findings; their study showed that the buccal positioning of IMC can increase the severity of ALRR. This difference can be due to Ardakani et al.'s smaller sample size (40 samples) or their sample's different age range (11–45 years).

The present study showed that an increase in the distance of the canine cusp tip from the occlusal plane decreases the probability of ALRR in the middle third of the root. Our team found no previous studies evaluating the relationship between the vertical position of IMC and the location of ALRR.

Similar to the studies of Hadler‐Olsen et al. (2015), Ucar et al. (2017), and Ardakani et al. (2021), results of our research showed no significant association between the severity and location of ALRR and the angle between the long axis of canine and long axis of lateral incisor (CAL), the angle between the long axis of canine and occlusal plane (CAO) and the angle between the long axis of canine and midline (CAM). Although Guarnieri et al.'s (2016) study in 2016 showed no significant relationship between ALRR and CAM and CAO, they reported that if the angle between the long axis of IMC and the adjacent lateral incisor is higher than 54°, the probability of ALRR exceeds by 61%.

According to the results of our study, the distance between the cusp tip of the IMC and the occlusal plane can be used as an index for early detection and prevention of ALRR, especially in female patients with IMC. If this distance is high in a patient, periodic examinations may be required to prevent the impaction conditions from deteriorating and to reduce the risk of ALRR to a minimum.

This study had limitations. Evaluating the changes in the severity of ALRR due to IMC in one person over time would have led to more reliable findings. Due to the hazards of X‐ray exposure in several imagings, this study could not be conducted with a longitudinal approach (Schroder et al. 2018; Ucar et al. 2017). Owing to the retrospective design of the study, the acquisition of supplementary CBCT images for more exact classifications of root resorption was not feasible. Recommendations for future research include the observation of the relationship between various axial IMC follicle positions (including the bicortical position) and the risk and extent of ALRR. In the present study, the Kurol–Ericson scale was used to measure the severity of ALRR, and their study was referred to only include the buccal/palatal position of the impacted canines (Alqerban et al. 2009). Therefore, it is recommended to use a more objective and detailed classification for root resorption degree, to incorporate measurements in all root aspects, such as the Three‐Dimensional Leeds Orthodontic Root Resorption Target Scale (3‐D‐LORTS) developed by Jawad et al. (2016). The measurement of the follicle size is also recommended to involve a volumetric, 3D assessment in different sections.

Within all these limitations, the number of affecting factors evaluated in this research makes its results potentially useful in clinical decision‐making and treatment planning regarding canine impaction in the Iranian population.

## 5 Conclusion

The main findings of this retrospective study were as follows:

- The prevalence ratio of maxillary canine impaction in the palatal position compared to the buccal position was 5:1.
- The severity of adjacent lateral root resorption had a significant relationship with sex and was higher in females.
- The distance between the impacted canine cusp tip and the occlusal plane was associated with the location of adjacent lateral root resorption. An increase in this distance decreased the probability of resorption in the middle third of the lateral incisor.

## Author Contributions

**Mahkameh Moshfeghi:** visualization, supervision, project administration. **Maryam Hosseini:** project administration, data curation, methodology. **Parastoo Iranparvar:** validation, data analysis, manuscript writing and editing. **Amirali Momeni:** project administration, data curation, methodology. **Negin Eslamiamirabadi:** validation, data analysis, manuscript writing. **Mina Biria:** supervision, project administration, manuscript editing.

## Funding

The authors have nothing to report.

## Disclosure

No artificial intelligence (AI) tools were used in the design, analysis, or writing of the manuscript.

## Ethics Statement

The study was approved by the Committee for Ethics in Research, School of Dentistry, Shahid Beheshti University of Medical Sciences (IR.SBMU.DRC.REC.1398.198).

## Consent

Data were collected using the archived CBCT documents of the department of oral and maxillofacial radiology, anonymously.

## Conflicts of Interest

The authors declare no conflicts of interest.

## Data Availability

The data that support the findings of this study are available from the corresponding author upon reasonable request.
